# The "Normyl Treatment" of Alcoholism

**Published:** 1912-05-25

**Authors:** Wm. Porteous

**Affiliations:** Secretary of the Normyl Treatment Association.


### The "Normyl Treatment" of Alcoholism.

*To the Editor of* The Hospital.

Sir,—My attention has been called to an article on the Normyl Treatment appearing in your issue of this week, and I should be glad if you would allow me the courtesy of a small space in your columns to make known to your many readers the other side of the question. In the first place, the analysis by the *British Medical Journal,* upon which your article is based, is admittedly neither a conclusive nor a complete analysis, so that the whole of your carefully-drawn-up argument falls to the ground, as it is founded upon an entirely inaccurate premise.

With your permission, I might point out that during the last seven years the Normyl Treatment Association has dealt with over 8,000 patients suffering from alcoholism, and we have undeniable proof that the great majority of these have been not only freed from the craving for alcohol, but also have been restored both mentally and physically. In addition, it must be remembered that the Normyl Treatment only occupies twenty-four days, and has succeeded in curing an infinitely larger percentage of alcoholic patients than any inebriate home or retreat in the United Kingdom, although in most cases the length of stay in such institutions exceeds six months. This statement I am willing to submit to any impartial tribunal.

In conclusion, I would add that before forming this Association my Committee satisfied themselves of the nature and efficacy of the treatment on the authority of a physician of the very highest standing, to whom it was submitted for analysis. Further, it is now recommended by over 400 doctors practising in the United Kingdom, and probably 40 per cent. of our patients come to us either directly or indirectly on the advice of their medical attendants. Apart from what the medical profession may say through its press, there is a certain eloquence of facts, and these facts I have much pleasure in submitting to your readers, so that they themselves may judge whether or no the Normyl Treatment is or is not a blessing to mankind.—Yours faithfully, Wm. Porteous,

Secretary of the Normyl Treatment Association.

May 11.

[To the minds of many besides ourselves, we believe, the most eloquent fact about this whole business is the presence of alcohol in a cure for drunkenness. Mr. Porteous does not deny it. Does the physician of high standing referred to? We shall be glad to have his name and full details of his analysis, and to learn whether this latter was carried out personally or by proxy. Although criticism here of an *ex-parte* statement must necessarily be brief, we must make space for the following point: The Normyl Treatment Association through its secretary speaks only of alcoholics, whereas the medicament used is described as potent also against drug addictions, presumably of all the various kinds commonly met with. Are then morphinomaniacs, for instance, treated with hourly doses of alcohol, and is "nipping" the only hope for the abuser of cocaine, chloral, hashish, paraldehyde, or whatever it be?—Ed., The Hospital.]

